# Changing cerebral blood flow in normal pressure hydrocephalus after the tap test can predict clinical improvement

**Published:** 2014-10-06

**Authors:** Behnaz Sedighi, Kaveh Shafiee, Rostam Seifaldini, As'ad Abdi

**Affiliations:** 1 Department of Neurology, School of Medicine, Kerman University of Medical Sciences, Kerman, Iran; 2 Department of Neurology, School of Medicine, Neurology Research Center, Kerman University of Medical Sciences, Kerman, Iran

**Keywords:** Normal Pressure Hydrocephalus, Cerebral Blood Flow, Transcranial Doppler Sonography

## Abstract

**Background:** We studied the role of cerebrospinal fluid (CSF) tap test at idiopathic normal pressure hydrocephalus (INPH) in improving cerebral blood flow velocity indices by transcranial Doppler (TCD) sonography.

**Methods:** Twelve patients with assumed INPH were included in the study. The CSF tap test and INPH grading score was carried out according to the standard protocol. TCD was performed before and after the tap test for assessing blood flow in middle cerebral and anterior cerebral arteries.

**Results:** Five INPH patients (41.7%) had clinical improvement as defined by at least one point reduction in INPH grading scale. The baseline TCD parameters of the middle cerebral artery were significantly higher compared with the control, and those parameters were decreased after tap test in those who improved.

**Conclusion:** Our study showed that improvement in INPH grading score after CSF tap test might correlate with changing in TCD parameter in MCA and TCD parameter might be useful for shunt response in these patients.

## Introduction

The idiopathic normal pressure hydrocephalus (INPH) syndrome is a common cause of potentially reversible dementia and diagnosed in 5-10% of a demented patient.^1^ INPH has the prevalence up to 181.7/100,000 for people 70-79 years of age.^2^

In general, dementia, gait apraxia, and sphincter dysfunction are required to consider the diagnosis. The improvement of any mentioned clinical impairments after lumbar cerebrospinal fluid (CSF) withdrawal or tap test offers a reliable means for selection of patients likely to respond to shunting in INPH.^3^

The CSF tap test fist has been introduced first by Hakim and Adams^4^ and then improved by Wikkelso et al.^5^ Wikkeslo et al. believed that improvement in cerebral blood flow (CBF) might have some role beside other factors in CSF tap test response.

Despite more than 60 years of research, our understanding of INPH pathophysiology remains sparse.^5^

Transcranial Doppler (TCD) sonography becomes a noninvasive technique for measurement of cerebral blood flow velocity (CBFV). Besides the mean flow velocity, dynamic changes in the pulsatility index (PI) and the resistance index (RI), as calculated from TCD data, allow for an assessment of the forces acting on the terminal vasculature of the brain.^6^

Hence, it is a useful and non-invasive tool to assess the cerebral hemodynamic. The aim of this study was to assess the TCD CBF parameters before and after the CSF tap test and compare it with the INPH grading scale.

## Materials and Methods

This was an uncontrolled before and after study. Between January 2008 and February 2009, 12 demented patients with clinical criteria of INPH and enlarged ventricular systems (Evan’s ratio >0.3) were enrolled.

Evan’s index is defined by the maximum width of the frontal horn divided by the maximum inner width of skull.^2^

A control group consisting of 12 patients with cerebral atrophy on CT and dementia of different etiologies was included to establish baseline TCD data.

CSF tap test was performed at the Neurology Department, Shafa Hospital, Kerman University of Medical Sciences, Kerman, Iran.

TCD was performed with trans-temporal window using a 2 MHz Doppler probe (multi-Doppler probes, Sipplingen, Germany) on both sides if possible. TCD was done by a neurologist blinded to the study protocol and parameters, including peak systolic velocity (PSV); end diastolic velocity (EDV); mean cerebral velocity (MSV); PI; and RI were recorded. CBFV in the middle cerebral artery was measured using TCD before and next day after CSF tap test.

Clinical state of study subjects before and after the CSF tap test were assessed by the INPH grading scale, which evaluates the three main parts of the NPH syndrome: gait, cognitive function, and sphincter disturbances (Table 1). It ranges from a score of 0-4 in each domain of disturbance of gait, cognition and urination. Improvement of clinical state was considered as positive if the patient’s performance had improved in at least one score.

The change of gait was evaluated 1 or 2 days after the tap while change of cognition and urination was evaluated at 1 week.

The CSF tap test was carried out according to Wikkelso et al. protocol^7^^,^^8^ on all study subjects on 2 consecutive days and at the same hour each day. On the morning of the 1^st^ day, the patients underwent evaluation of gait and cognitive functions. On the 2^nd^ day, these evaluations were repeated, 2 and 8 h after a lumbar puncture (LP) with removal of at least 50 ml of CSF.

We had analyzed normality of our data and according to the Shapiro–Wilk test, although it had small sample size, but its distribution was normal. Hence, we could use mean and standard deviation for our analysis.

The groups were compared using the Student’s t-test or Mann–Whitney U test for the continuous variables. Measures of TCD parameters before and after the tap test were compared using paired t-test. P-values of 0.05 or less were considered as statistically significant.

## Results

INPH patient’s mean age was 67 years and 58.3% of them were male.

We compared the MCA’s mean flow velocity of our INPH patient with the control group at the baseline. The mean flow velocity was nearly 43 cm/s in each group.

Table 2 shows the TCD parameter in the INPH patients before and after the CSF tap test. The mean flow velocity did not significantly change after CSF tap test in the MCA.

**Table 1:** Idiopathic normal pressure hydrocephalus grading scale

**Cognitive impairment**	
0	Normal
1	Complaints of amnesia or inattention but no objective memory and attentional impairment
2	Existence of amnesia or inattention but no disorientation of time and place
3	Existence of disorientation of time and place but conversation is possible
4	Disorientation for the situation or meaningful conversation impossible
Gait disturbance	
0	Normal
1	Complaints of dizziness of drift and dysbasia but no objective gait disturbance
2	Unstable but independent gait
3	Walking with any support
4	Walking not possible
Urinary disturbance	
0	Normal
1	Urinary urgency
2	Occasional urinary incontinence (1-3 or more times per week but less than once per day)
3	Continuous urinary incontinence ( 1-3 or more times per day)
4	Bladder function is almost or completely deficient

**Table 2:** Middle cerebral artery’s transcranial Doppler sonographic indexes before and after cerebrospinal fluid tab test

**MCA index at: **	**Mean velocity**	**PSV**	**PI**	**RI**
Base line	43	76.3	1.1	0.6
After CSF tap	41.3	71	1.0	0.4

MCA: Middle cerebral artery; PSV: Peak systolic velocity; PI: Pulsatility index; RI: Resistance index; CSF: Cerebrospinal fluid

Following CSF tap test, five INPH patients (41.7%) had clinical improvement as defined by at least one point reduction in INPH grading scale.

Interestingly, we observed that in those who improved after CSF tap test, the baseline TCD parameters of the middle cerebral artery (PSV, EDV, and MCV) were significantly higher compared to the control, and those parameters were decreased after tap test. Those changes were not statistically significant for anterior cerebral artery (ACA) as has presented in table 3.

**Figure 1:**
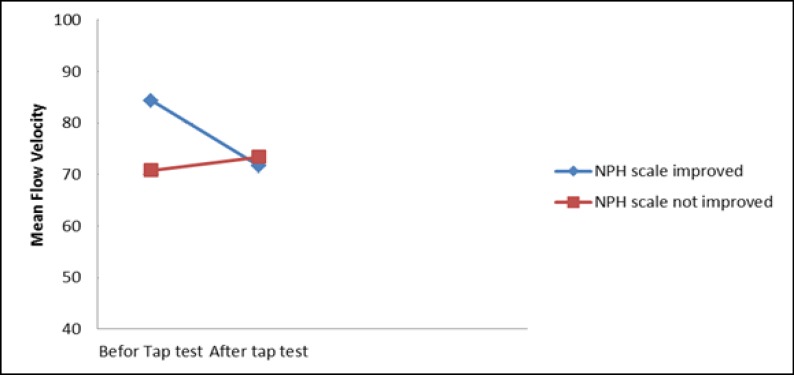
Changing middle cerebral mean flow velocity compared to improvement in NPH scale after tap test

As shown in table 3 and figure 1, MCA’s mean flow velocity in the INPH patient whose INPH scale score improved after the CSF tap test was 84.4 cm/s, which was significantly higher as compared with the patients who had not improve (70.8 cm/s). That parameter significantly had reduced to 71.7 cm/s after the CSF tap test.

## Discussion

Our study showed that improvement in INPH grading score after CSF tap test might correlate with changing in TCD parameter in MCA and TCD parameter might be useful for shunt response in these patients.

The hemodynamic abnormality in INPH patients is not a new findings. Greitz and his colleagues showed than CBF changes after CSF shunting in INPH patients.^9^ Mathew, and his colleagues recommended that assessing CBF changes could assist in both diagnosis and selection of patients for CSF shunting. They observed that regional CBF and velocity reduced in the territory of the ACA in INPH compared to dementia due to brain atrophy. In their study, CBF increased after lowering the CSF pressure by LP in patients with INPH. Patients with more increases in rCBR after lowering CSF pressure showed the most consistent clinical improvement after CSF shunting.^10^

After them, many studies also pointed out the correlation between changing in intracranial pressure (ICP) and CBFV. Increased CBF after shunting has been proposed as responsible for the clinical improvement in patients with INPH.^10^

**Table 3:** Cerebral blood flow indices before and after the tap test related to clinical improvement in idiopathic normal pressure hydrocephalus patients

**Cerebral blood flow index**	**Before tap test**			**After tap test**		
	**INPH scale changed**	**INPH scale not changed**	**P**	**With improvement**	**Without improvement**	**P**
Middle cerebral artery						
Peak systolic velocity	84.4 ± 4.6	70.8 ± 4.4	< 0.001	71.7 ± 6.1	73.4 ± 5.9	0.638
End diastolic velocity	31.2 ± 3.3	25.3 ± 3.4	0.013	25.9 ± 3.6	27.5 ± 3.8	0.480
Mean cerebral velocity	48.8 ± 3.9	40.6 ± 2.9	0.002	40.1± 4.4	42.5 ± 4.7	0.392
Anterior cerebral artery						
Peak systolic velocity	63.3 ± 4.4	59.7 ± 5.5	0.320	65.2 ± 5.7	60.1 ± 5.0	0.131
End diastolic velocity	22.9 ± 3.1	20.2 ± 3.7	0.214	22.7 ± 2.4	20.0 ± 2.1	0.065
Mean cerebral velocity	35.5 ± 3.5	33.3 ± 2.9	0.261	38.8 ± 3.5	34.3 ± 2.8	0.101

Data are presented as mean ± SD. SD: Standard deviation; INPH: Idiopathic normal pressure hydrocephalus

It is also previously showed that even moderate ICP changes could limit the modulation of CBF, and that revealed a direct interaction between the CSF space and the cerebrovascular compartment.^11^ This prospective study also shows that MCA blood flow decreases in INPH patients who improve after tap test. A small number of cases prevent a strong clinical pronouncement, but our results are sufficient in showing correlation between clinical improvement and higher MCA blood velocity in INPH patients.

On the other hand, there are many studies that did not find any significant changes in cerebral hemodynamic after shunting in the INPH patients.^12^^-^^14^

Our findings are more comparable to a study by Bakker and his colleagues. They showed similar results before and after shunting these patients and concluded that higher cerebral blood velocity before surgery in the INPH patients was related to clinical improvement.^15^ Traczewski et al. also suggested that restoration of cerebral perfusion after CSF withdrawal was associated with a high likelihood of shunt success.^3^

The pathophysiological mechanism underlying clinical improvement after CSF tapping in INPH has not been defined clearly yet. It may include restoration of regional blood flow, or metabolic improvement.

Chang has shown that microcirculation and pressure exerted on the nerve fibers in the frontal lobe is closely correlated with dementia in INPH.^14^

In our study it seems that, the MCA hemodynamic and INPH grading score are correlated. We have showed that, in INPH patients if MCA flow parameter does not change, his grading scale will also not too.

As said before many of our patients did not respond to CSF tap. In this group of patients, the TCD parameters also did not significantly change. Based on the Kondziella’s hypothesis, there is a certain “point of no return” in pathogenesis of INPH from CSF dynamics. This is probably the reason why despite restored CSF circulation by CSF tap or shunting many patients with chronic hydrocephalus still suffer from severe neurological deficits and did not improve.^1^

## Conclusion

Our study suggests changing in CBF in patients with INPH after CSF tap may have a role in clinical improvement.

Our study had many limitations:

First, there are no accurate tests or diagnostic criteria to diagnose the INPH. The available diagnostic tools have limited sensitivity, and specificity and diagnosis is still largely based on measuring CSF dynamics.

Recently, the reliability of CSF tap test has been evaluated by Wikkelso et al. They reported that the positive predictive value the tests was >90% and the negative predictive value <20%. Overall accuracy of CSF tap test was 53%. They also concluded that the CSF tap test did not correlate with outcome in INPH after 12 months. According to them, CSF tap test can be used for selecting patients for shunt surgery but not for excluding patients from treatment.^8^

Second, nearly 10% of the patients had closed trans-temporal window that did not let us study intracranial artery by TCD.

Finally, larger studies that include more INPH patients should be recommended on this topic.

## Notes

**How to cite this article: **Sedighi B, Shafiee K, Seifaldini R, Abdi A. Changing cerebral blood flow in normal pressure hydrocephalus after the tap test can predict clinical improvement. Iran J Neurol 2014; 13(4): 245-9.
